# Retraction: The Carboxy-terminus of BAK1 regulates kinase activity and is required for normal growth of Arabidopsis

**DOI:** 10.3389/fpls.2016.00960

**Published:** 2016-06-24

**Authors:** Frontiers Editorial Office

The Journal and Authors retract the 4 February 2014 article cited above for the following reasons provided by the Authors:

After publication of this article, we became aware that the transgenic plants described in this manuscript were not correct because of inadvertent errors in genotyping of the parent plants, and thus the results presented in Figures 4, 6 are not valid. We realized from the immunoblot result presented in Figure 4B, that immunoprecipitated BAK1-ΔCT-Flag had the same apparent molecular weight as did BAK1-Flag, despite the fact that truncated protein should have been ~4-kDa smaller but that size difference was not apparent. We subsequently confirmed that the BAK1 construct used was not truncated but was full length. At the moment we cannot explain why the transgenic plants show reduced growth, but it is clear that it is not related to the BAK1 transgene. However, the results presented in Figures 1–3, 5 with recombinant proteins *in vitro* are valid, and thus conclusions drawn from functional and interaction studies *in vitro* are correct. Regrettably, the relevance to the function of BAK1 *in vivo* remains uncertain and it is no longer clear whether the carboxy-terminus of BAK1 is “required for normal growth of Arabidopsis” as stated in the title. The responsibility for this experimental error rests with only two of the authors: the senior author (MHO) who was a Senior Research Scientist when these studies were conducted with extensive experience in molecular biology and whose primary responsibility was production of the transgenic plants. The other responsible individual was the corresponding author (SCH), who failed to provide adequate oversight to the production of the transgenic plants and as a result, critical checks and balances were not in place. The other authors (WX, SYK, XW, and SDC) are not responsible in any way for this regrettable situation. We are pursuing follow-up experiments to correctly generate the BAK1-ΔCT-Flag transgenic plants in the *bak1 bkk1* double null background that are required to test the functional role *in vivo* and hope to publish soon.

We sincerely regret any scientific misconceptions that have been caused by the above paper; the corresponding author's laboratory wasted an additional year studying these plants before the error was discovered. We apologize to the entire scientific community for the misinformation and any adverse consequences that may have resulted.

